# Pathways in the Diagnosis and Management of Diabetic Polyneuropathy

**DOI:** 10.1007/s11892-015-0609-2

**Published:** 2015-04-22

**Authors:** Michelle Kaku, Aaron Vinik, David M. Simpson

**Affiliations:** Department of Neurology, Icahn School of Medicine at Mount Sinai, New York, NY USA; Eastern Virginia Medical School, Strelitz Diabetes Research Institute, 855W. Brambleton Avenue, Norfolk, VA 23510 USA

**Keywords:** Distal symmetric polyneuropathy, Diabetic, Neuropathy, Diagnosis, Treatment algorithm, Multidisciplinary

## Abstract

Distal symmetric polyneuropathy (DSPN), the most common form of diabetic neuropathy, has a complex pathophysiology and can be a major source of physical and psychologic disability. The management of DSPN can be frustrating for both patient and physician. This article provides a general overview of typical patient pathways in DSPN, and highlights variations in diagnosis, management, and referral patterns among different providers. DSPN is managed in several settings by primary care physicians (PCPs), specialists, and nurse practitioners. The initial clinical management of the patient is often dependent on the presenting complaint, the referral pattern of the provider, level of comfort of the PCP in managing diabetic complications, and geographic access to specialists. The primary treatment of DSPN focuses mainly on glycemic control and adjustment of modifiable risk factors, but other causes of neuropathy should also be investigated. Several pharmacologic agents are recommended by treatment guidelines, and as DSPN typically exists with comorbid conditions, a multimodal therapeutic approach should be considered. Barriers to effective management include failure to recognize DSPN, and misdiagnosis. Patient education also remains important. Referral patterns vary widely according to geographic location, access to services, provider preferences, and comfort in managing complex aspects of the disease. The variability in patient pathways affects patient education, satisfaction, and outcomes. Standardized screening tools, a multidisciplinary team approach, and treatment algorithms for diabetic neuropathy should improve future care. To improve patient outcomes, DSPN needs to be diagnosed sooner and interventions made before significant nerve damage occurs.

## Introduction

Peripheral neuropathy affects 26 % to 47 % of people with diabetes in the USA [1]. The most common form of diabetic neuropathy is distal symmetric polyneuropathy (DSPN), occurring in up to 50 % of patients with neuropathy. The pathophysiology of DSPN is complex and its management can be frustrating for both the patient and the physician. DSPN can be a major source of disability, both physically and psychologically, and is an independent risk factor for depressive symptoms [2]. High pain levels are associated with poor sleep, functioning, and productivity [3]. Direct and indirect costs, including prescription medications and office visits, are also significantly higher among patients with greater pain severity [3].

The estimated annual cost of diabetic neuropathy and its complications in the USA in 2003 was between $4.6 and $13.7 billion [4]. Although diabetic neuropathy is often thought of as an adult disease, neuropathy has been reported in 11 % of youths with type 1 diabetes, and may occur sooner after diagnosis in children with type 2 diabetes [5, 6]. This review assesses typical patient pathways in DSPN and identifies variations in diagnosis, management, and referral patterns among different providers.

## Materials and Methods

Literature searches were carried out from the beginning of 2010 to June 2014 to identify published evidence on DSPN and its management. PubMed was searched using the terms ‘(diabetic OR diabetes) AND (neuropathy OR pain)’ while congresses of the European Association for the Study of Diabetes, American Diabetes Association, and International Diabetes Federation were searched using ‘neuropathy’, ‘pain’, ‘PDN’, and ‘DSPN’. All abstract titles were assessed for papers of relevance.

### Types of Neuropathy

There are typical and atypical forms of DSPN [7, 8]. The Toronto Consensus Panel on Diabetic Neuropathy defined typical DSPN as a “chronic, symmetrical, length dependent sensorimotor polyneuropathy” [9]. Atypical DSPN has a monophasic or fluctuating course and may have asymmetric or proximal symptoms, as well as motor involvement. Acute painful DSPN has been characterized as an additional subtype that presents predominantly with pain, particularly sharp, stabbing, and electric-shock sensations in the distal extremities that may include nocturnal exacerbations [10]. Such painful small-fiber neuropathy, with minimal objective neurologic signs, may occur in prediabetes [11]. Other atypical forms of neuropathy that occur in diabetes include focal and multifocal neuropathies, such as mononeuropathies, cranial neuropathies, plexopathies, radiculopathy, mononeuritis multiplex, amyotrophy, predominantly small-fiber neuropathy, and autonomic neuropathy. Chronic inflammatory demyelinating neuropathy is also more common in diabetic than in non-diabetic patients [12].

### Symptoms and Clinical Features

The most common symptoms of DSPN are length dependent, usually affecting the feet first and progressing proximally. Symptoms are predominantly sensory and can be classified as “positive” (tingling, burning, and other abnormal sensations) or “negative” (sensory loss, weakness, numbness, and unsteady gait). Painful DSPN is often described as burning or electric and tends to occur more often at night. Motor symptoms are less common but can occur later in the disease course. Distal deep-tendon reflexes are typically reduced or absent.

The most serious complications of DSPN include foot ulcers, Charcot foot abnormalities, injuries, and ultimately, lower-extremity amputation, especially when concomitant peripheral vascular disease causes foot ischemia. Degradation of sensory function leading to imbalance and unsteadiness in gait [13] with loss of proprioception results in increased likelihood of a fall [14]. Decreased sensation of the distal extremities makes small injuries and ulcers common, and more than 2 % of patients with diabetes develop new foot ulcers each year [15]. The lifetime risk that a patient with diabetes will acquire a foot lesion, including an ulcer or gangrene, is estimated to be approximately 15 % to 25 % [16]. The chronic nature of DSPN can lead to anxiety, depression, catastrophizing behavior, an inability to accept chronic pain, and sleep disturbances [17].

### Risk Factors for DSPN

The most important risk factor for developing DSPN in type 1 diabetes is poor glycemic control. In the Diabetes Control and Complications Trial, intensive therapy reduced development of clinical neuropathy by 64 % compared with standard glucose control over 5 years [18]. In the Epidemiology of Diabetes Interventions and Complications follow-up study, the benefits of intensive insulin treatment persisted for 14 years, despite similar glycemic control between the groups following study completion [19, 20••]. The EURODIAB IDDM Complications Study found additional correlations between neuropathy and duration of type 1 diabetes, quality of metabolic control, age, height, cigarette smoking, high-density lipoprotein cholesterol, proliferative diabetic retinopathy, and cardiovascular disease [21].

Risk factors for DSPN in type 2 diabetes are similar to the risks for vascular disease, such as smoking, obesity, hyperlipidemia, age, and waist circumference. Many of these risk factors are modifiable, highlighting the importance of patient self-motivation and the potential influence of physician counseling in determining disease progression. Among prediabetic patients, increased fasting glucose and impaired glucose tolerance are associated with a high risk of clinical DSPN, comparable with that of diabetic patients [22, 23], highlighting the need for early therapeutic intervention. In patients with neuropathy associated with impaired glucose tolerance, partial cutaneous re-innervation is possible through improvements to diet and exercise after counseling [24]. The United Kingdom Prospective Diabetes Study found a reduced risk of neuropathy with intensive treatment compared with standard glycemic control [25]. This reduced risk must be weighed against the potential risk of overly aggressive glycemic control, which may be associated with acute painful DSPN [26] and increased cardiovascular risk and sudden death related to autonomic dysfunction [27–29].

### Pathophysiology

The pathophysiology of DSPN is not fully understood and is likely multifactorial. Nerve biopsy from patients with painful neuropathy indicates that there is degeneration of myelinated and unmyelinated fibers [30, 31]. Metabolic derangements have been implicated, such as oxidative and nitrosative stress, accumulation of glycation end products, impaired calcium homeostasis, and mitochondrial dysfunction [32–34], and increased activity through the polyol pathway [35]. Impaired insulin signaling may directly injure the dorsal root ganglia and play a role in the pathogenesis [36]. The mechanisms involved in metabolic syndrome may contribute to a self-perpetuating cycle of oxidative and nitrosative stress, inflammatory signals, and disruption of normal cellular function [37•, 38•]. Peripheral lesions may also have central effects, particularly through central sensitization of nociceptive neurons [39].

### Diagnosis

Diagnosis of DSPN is primarily clinical and involves a thorough history and physical examination with a focus on cardiovascular and neurologic tests, and a detailed assessment of the feet [40]. Early diagnosis of DSPN is imperative in preventing irreversible damage; however, 50 % of patients may be asymptomatic. A 1-g Semmes–Weinstein monofilament is useful for detecting changes in sensitivity [41], and a 10-g monofilament is useful for predicting ulcer risk. A small decrease in the duration of a vibratory stimulus sensation, assessed with a 128-Hz tuning fork, is an early indicator of neuropathy. A more quantitative vibration assessment is available using the Rydel–Seifer tuning fork. The hallux, as opposed to the fifth metatarsal head, is a more sensitive indicator of neuropathy in patients with diabetes [42]. A careful examination of the foot should include a check for peripheral pulses, to assess for peripheral vascular disease, and a visual check for ulcers. As painful diabetic peripheral neuropathy (pDPN) is usually symmetrical, patients with asymmetrical symptoms or signs should be carefully assessed for other etiologies of their symptomatology [9].

Nerve conduction studies (NCS) often form part of the evaluation of DSPN, especially in atypical cases with superimposed nerve entrapment or inflammatory demyelinating neuropathy and in patients with minimal or no objective neurologic signs. While NCS are helpful in diagnosing patients with large-fiber neuropathy, they have limited utility in diagnosing small-fiber neuropathy. Small-fiber function may be assessed by skin biopsy and quantitation of intra-epidermal nerve fiber density, particularly when results of NCS are normal. Skin biopsy is a minimally invasive procedure [43–46]. Decreased intra-epidermal nerve fiber density is indicative of small-fiber neuropathy. Further imaging, such as computed tomography or magnetic resonance imaging, is usually not necessary unless there is clinical suspicion for nerve entrapment or disc pathology.

As DSPN is a diagnosis of exclusion, other etiologies of polyneuropathy should also be assessed: alcohol use; vitamin B12 levels; vasculitis; serum protein electrophoresis and immunofixation; infections (e.g., Lyme disease, HIV); and cancer and related paraneoplastic syndromes [47]. For example, patients with vitamin B12 deficiency have impaired sensory and motor peripheral nerve function [48]. Notably patients may have functional consequences of vitamin B12 deficiency even with levels in the “low normal” range, and should receive supplementation with methylcobalamin [48]. Metformin may contribute to vitamin deficiency [49].

The Toronto Consensus Panel on Diabetic Neuropathy defined specific diagnostic guidelines that estimate the severity of DSPN based on NCS and various signs and symptoms [50]. Additionally, questionnaires are frequently used to identify and quantify neuropathy including the Michigan Neuropathy Screening Instrument [51], the McGill Pain Questionnaire [52], the Neuropathic Pain Questionnaire [53], the Brief Pain Inventory [54], the Neuropathic Pain Symptom Inventory [55], the Norfolk Quality of Life Questionnaire-Diabetic Neuropathy Questionnaire [56], and the Neuropathy and Foot Ulcer-specific Quality of Life Instrument [57]. Standardized screening tools provide a good clinical record for post-treatment follow-up, are simple to use, and are easily administered by a physician assistant, nurse practitioner, or self-completed by the patient before the office visit [58].

### Treatment

The focus of DSPN management is disease modification and symptomatic relief; no treatment completely prevents or reverses disease progression. Pancreas transplants [59], diet and exercise [24], and topiramate [60] have all been shown to induce small-fiber regeneration. Rational glycemic control is the primary approach to manage symptoms and prevent further damage, including falls and foot ulcers. Most clinical trials have studied therapies for symptomatic pain relief. Although various treatment approaches are recommended by diabetic and national societies, this review focuses on pharmacologic agents for symptomatic treatment. Duloxetine, pregabalin, and tapentadol are Food and Drug Administration (FDA)-approved medications for DSPN, although many other agents have been studied and are frequently used. To achieve an optimal therapeutic outcome, it is important to identify and treat any comorbid conditions. Some treatments may improve pain and sleep by direct and indirect pathways. Many treatments for DSPN require careful dose titration every 2–4 weeks based on efficacy and safety. Combinations may also be useful, although consideration of potential drug–drug interactions is important, and combining first-line agents is not backed by trial evidence [61•].

Various organizations, professional societies, and expert panels have produced guidelines for the treatment of neuropathic pain including DSPN, such as the Toronto Consensus Panel on Diabetic Neuropathy [61•], the Neuropathic Pain Special Interest Group (NeuPSIG) [62], the European Federation of Neurological Societies Task Force [63], the National Institute for Health and Care Excellence (NICE) [64], the American Association of Neurology (AAN) in collaboration with the American Association of Neuromuscular and Electrodiagnostic Medicine and the American Academy of Physical Medicine and Rehabilitation [65], the Working Group on the Diabetic Foot from the French-Speaking Society of Diabetology [66], and the American Association of Clinical Endocrinologists [67]. Several of these were compared in a review by Spallone [68]; guidelines generally recommend considering tricyclic antidepressants (TCAs), serotonin/norepinephrine-reuptake inhibitors (SNRIs), and alpha-2-delta ligands as first-line agents. Many guidelines also recommend duloxetine as a first-line option.

### Tricyclic Antidepressants

TCAs are commonly used agents for DSPN and their analgesic effect is likely to be mediated through a different pathway from their antidepressant effect. Anticholinergic and cardiac side effects are the biggest limitation to their use. Imipramine and desipramine have a lower side-effect burden than amitriptyline. The NeuPSIG guideline recommends TCAs as first-line agents, although they urge caution when using them in patients with ischemic cardiac disease or ventricular conduction abnormalities, suggesting a screening electrocardiogram in patients aged ≥ 40 years and limiting doses to < 100 mg/day [69]. The NICE guideline focuses on pharmacologic recommendations in the non-specialist setting and also includes amitriptyline among their list of first-line agents [64]. The clinical characteristics of these TCAs are summarized in Table 1.

**Table 1:** Summary of tricyclic antidepressants as potential treatment options for diabetic peripheral neuropathy [63–65, 69, 70]

Treatment	Mechanism of action	Advantages	Disadvantages	NNT (95 % CI)	NNH (95 % CI)
*TCAs*				2.1 (1.9–2.6)	15.9 (11–26)
Amitriptyline	Inhibition of reuptake of monoamine neurotransmitters (serotonin and norepinephrine) from presynaptic terminals and blockade of ion channels	• Efficacious in pDPN • Beneficial for patients with comorbid depression • Sleep enhancing • Generic medication • First-line agent • Healthcare professional familiarity from other indications • Inexpensive	• Not FDA-approved for pDPN • Cholinergic and cardiac side effects • Caution for patients with ischemic cardiac disease/ventricular conduction abnormalities • ECG • Other side effects include constipation, incontinence, dry mouth, dizziness, drowsiness, cognition deterioration • Unpredictable patient response • Lengthy titration required • Caution indicated for coadministration with SSRIs • Drug–drug interactions with CYPD6 inhibitors • Contraindicated for concomitant use with MAOIs		
Desipramine	As for amitriptyline, but with a relatively reduced effect on serotonin	• Better side-effect profile than amitriptyline	• As for amitriptyline		
Imipramine	Active metabolite is desipramine, following conversion by the liver	• Better side-effect profile than amitriptyline	• As for amitriptyline		

*CI* confidence interval, *CYP* cytochrome P450, *ECG* electrocardiogram, *FDA* Food and Drug Administration, *MAOI* monoamine oxidase inhibitor, *NNH* the number of patients needed to harm for one drop-out due to adverse events, *NNT* estimated number of patients with painful polyneuropathy needed to treat to achieve one patient with a 50 % reduction in pain, *pDPN* painful diabetic peripheral neuropathy, *SSRI* selective serotonin-reuptake inhibitor, *TCA* tricyclic antidepressant

### Serotonin/Norepinephrine-Reuptake Inhibitors

SNRIs, such as duloxetine and venlafaxine, regulate descending inhibitory pain pathways by inhibiting the reuptake of serotonin and norepinephrine. In several clinical trials, duloxetine has been shown to be efficacious for up to a year [71]. The most common adverse effects of duloxetine include nausea, whereas for venlafaxine they are gastrointestinal disturbances. The NeuPSIG guideline recommends SNRIs as first-line agents. They suggest caution in patients with cardiac disease and advise a tapering schedule upon discontinuation of the drug to prevent withdrawal [69]. The NICE guideline recommends duloxetine as a first-line option; however, venlafaxine is not recommended [64]. The AAN guideline concludes that existing data are insufficient to recommend amitriptyline, venlafaxine, or duloxetine over one another [65]. The clinical characteristics of these SNRIs are summarized in Table 2.

**Table 2:** Summary of serotonin/norepinephrine-reuptake inhibitors as potential treatment options for diabetic peripheral neuropathy [63–65, 69, 70]

Treatment	Mechanism of action	Advantages	Disadvantages	NNT (95 % CI)	NNH (95 % CI)
*SNRIs*				5.0 (3.9–6.8)	13.1 (9.6–21)
Duloxetine	Inhibition of reuptake of the neurotransmitters serotonin and norepinephrine	• FDA-approved for pDPN • Efficacious in reducing pain scores for up to 1 year (efficacy beyond 12 weeks has not been systematically studied) • Improved tolerability vs TCAs • Beneficial for patients with comorbid depression • Simple dosing; effective dose is starting dose • Once-a-day dosing • First-line agent	• Tapering schedule necessary on discontinuation • Side effects include nausea, vomiting, dry mouth, constipation, somnolence, dizziness, decreased appetite, hyperhidrosis, sexual dysfunction • Potential for interactions due to metabolism via hepatic CYP1A2 and CYP2D6 enzymes • Contraindicated for use with MAOIs • May worsen glycemic control in patients with diabetes • Discontinuation rates of 15 %–20 % • Rare cases of hepatotoxicity		
Venlafaxine	As for duloxetine, but with a relatively reduced effect on norepinephrine at lower doses	• Efficacious in pDPN • May be added to gabapentin to improve response • Improves QoL measures • No dosage adjustment necessary if co-administered with a CYP2D6 inhibitor	• Not FDA-approved for pDPN • Requires titration schedule and tapering on discontinuation • Most common side effects include asthenia, sweating, nausea, constipation, anorexia, vomiting, somnolence, dry mouth, dizziness, nervousness, anxiety, tremor, blurred vision, and sexual dysfunction • ECG changes during treatment – should be prescribed with caution for patients with cardiac disease • Dose-dependent sustained blood pressure increases in some patients • Potential for drug–drug interactions with CYP3A4 inhibitors		

*CI* confidence interval, *CYP* cytochrome P450, *ECG* electrocardiogram, *FDA* Food and Drug Administration, *MAOI* monoamine oxidase inhibitor, *NNH* the number of patients needed to harm for one drop-out due to adverse events, *NNT* estimated number of patients with painful polyneuropathy needed to treat to achieve one patient with a 50 % reduction in pain, *pDPN* painful diabetic peripheral neuropathy, *QoL* quality of life, *SNRI* serotonin/norepinephrine-reuptake inhibitor, *TCA* tricyclic antidepressant

### Anticonvulsants

Anticonvulsants have a long history in the treatment of neuropathic pain. However, studies are sparse and results are inconsistent. Carbamazepine, oxcarbazepine, and lamotrigine block sodium channels and reduce neuronal excitability in the peripheral and central nervous system. Carbamazepine was one of the first antiepileptic drugs studied and had some success in reducing pain in several small studies [72, 73]. The most common side effects include dizziness, ataxia, sedation, hyponatremia, blurred vision, and confusion in the elderly.

Some studies of lamotrigine report significant relief of pDPN [74, 75], while others have failed to show any significant benefit either as monotherapy [76] or as an adjunctive treatment [77]. The most concerning, albeit uncommon, side effect of lamotrigine is Stevens–Johnson syndrome, whereas more common side effects include sedation, dizziness, and ataxia.

The AAN guidelines conclude that sodium valproate should be considered for the treatment of peripheral diabetic neuropathy, whereas lamotrigine, oxcarbazepine, and lacosamide should probably not be considered [65]. They also conclude that there is insufficient evidence to support or refute the use of topiramate. However, some evidence suggests that topiramate can induce skin intra-epidermal nerve fiber regeneration and enhance neurovascular function [60].

Pregabalin and gabapentin are active at the alpha-2-delta subunit of calcium channels; they decrease calcium influx, thereby decreasing central sensitization [63, 70]. As they are eliminated through the kidney and not the liver, the risk of drug–drug interactions is minimized. Both medications require titration schedules, and side effects include somnolence, dizziness, weight gain, headache, dry mouth, and peripheral edema. Pregabalin is the only medication that was given a level A recommendation by the AAN guidelines [65]; gabapentin was given a level B recommendation. Pregabalin and gabapentin are also both recommended as initial treatments for neuropathic pain in the NICE guidelines [64]. Improvements in patient function and quality of life in response to pregabalin treatment are correlated with the extent of pain relief [78]. However, rather than being mediated solely through pain relief, these improvements may also result from a combined effect on pain and sleep disturbance and a direct effect on patient function. The clinical characteristics of these anticonvulsants are summarized in Table 3.

**Table 3:** Summary of anticonvulsants as potential treatment options for diabetic peripheral neuropathy [63–65, 69, 70]

Treatment	Mechanism of action	Advantages	Disadvantages	NNT (95 % CI)	NNH (95 % CI)
*Anticonvulsants*				3.7 (2.6–6.4)	6.6 (4.9–10.0)
Carbamazepine	Blockage of voltage-gated sodium channels	• Efficacious for pDPN in small studies	• Not FDA-approved for pDPN • Efficacy limited/not evident in large placebo-controlled studies of DSPN • Common side effects include dizziness, ataxia, sedation, hyponatremia, blurred vision, and confusion in the elderly		
Lamotrigine	As for carbamazepine	• No clear advantages have been demonstrated for lamotrigine	• Not FDA-approved for pDPN • Limited evidence of efficacy • Common side effects include sedation, dizziness, and ataxia • Rare side effect of Stevens–Johnson syndrome		
Pregabalin	Active at the alpha-2-delta subunit of calcium channels and decrease calcium influx	• FDA-approved for pDPN • Efficacious in several neuropathic pain conditions • Dose-dependent effects • Improves QoL scores • Improved tolerability over older anticonvulsants (e.g., carbamazepine) • First-line agent • Metabolized via the kidneys not the liver so reduced potential for drug–drug interactions	• Requires titration schedule • Side effects include somnolence, dizziness, weight gain, headache, dry mouth, and peripheral edema • Dosage reduction necessary in patients with renal insufficiency • Discontinuation rates of 0 %–20 %	4.5 (3.6–5.9)	10.6 (8.7–14)
Gabapentin	As for pregabalin	• Efficacious in several neuropathic pain conditions but effect size is small • Improved tolerability over older anticonvulsants (e.g., carbamazepine) • First-line agent • May provide faster analgesia than pregabalin • Metabolized via the kidneys not the liver so reduced potential for drug–drug interactions • Generics available • Inexpensive	• Not FDA-approved for pDPN • Requires titration schedule • Side effects include dizziness, somnolence, and ataxia • Dosage reduction necessary in patients with renal insufficiency • Adequate trial of treatment can take ≥ 2 months	6.4 (4.3–12)	32.5 (18–222)

*CI* confidence interval, *FDA* Food and Drug Administration, *NNH* the number of patients needed to harm for one drop-out due to adverse events, *NNT* estimated number of patients with painful polyneuropathy needed to treat to achieve one patient with a 50 % reduction in pain, *pDPN* painful diabetic peripheral neuropathy, *QoL* quality of life

### Opioids

Chronic opioid use can lead to tolerance, dependence, constipation, and rebound headaches. Tramadol has a low affinity for μ-receptors and is a weak inhibitor of norepinephrine and serotonin reuptake, and moderately relieves DSPN-associated pain [79, 80]. Its side-effect profile includes constipation, sedation, and nausea. Tramadol has a lower potential for abuse than many opioids, although it can also lower the seizure threshold. Tapentadol, an FDA-approved agent for painful DSPN, combines a dual mechanism of action in a single formulation by combining an opioid agonist and a norepinephrine antagonist, which provides effective analgesia in patients with DSPN [81–83].

The NeuPSIG guidelines suggest opioids should be reserved for patients who do not respond to first-line medications, although they are recommended for acute neuropathic pain, neuropathic pain due to cancer, episodic exacerbations of severe neuropathic pain, and if necessary when titrating one of the first-line agents [69]. The AAN guidelines suggest that morphine sulfate, tramadol, and oxycodone controlled-release are probably effective in lessening the pain of DSPN [65]. An ultra-rapid acting fentanyl effervescent buccal tablet provides rapid relief of breakthrough pain in patients with diabetic and other forms of neuropathic pain [84].

Several studies indicate that rational combination therapy improves efficacy versus monotherapy without significantly increasing adverse effects [85–88]. For example, gabapentin plus long-acting morphine sulfate appears to be superior to either drug alone [87]. Prolonged-release oxycodone appears to enhance the analgesic effects of gabapentin [86], although low-dose oxycodone does not appear to improve analgesia with pregabalin [88]. Tramadol plus acetaminophen appears to provide comparable pain relief to gabapentin alone [85]. The clinical characteristics of these opioids are summarized in Table 4.

**Table 4:** Summary of opioid agents as potential treatment options for diabetic peripheral neuropathy [63–65, 69, 70, 81–83]

Treatment	Mechanism of action	Advantages	Disadvantages	NNT (95 % CI)	NNH (95 % CI)
*Opioids*			• Long-term use can result in tolerance, constipation, and rebound headaches	2.6 (1.7–6.0)	17.1 (9.9–66)
Tramadol	Weak opioid μ-receptor agonist Inhibits reuptake of serotonin and norepinephrine	• Efficacious in several neuropathic pain conditions including pDPN • Relatively rapid pain relief • Once-daily formulation • Generics available • Lower potential for abuse than other opioids • Combination treatment with acetaminophen appears better tolerated	• Not FDA-approved for pDPN • Limited efficacy • Side effects include constipation, sedation, and nausea • Lower threshold for seizures than other opioids • Used with caution in elderly patients due to risk of confusion • Not recommended for concomitant use with drugs acting on serotonin uptake (e.g., SSRIs)	4.9 (3.5–8.0)	13.3 (8.8–27)
Tapentadol ER	Weak opioid μ-receptor agonist Inhibits reuptake of norepinephrine	• FDA-approved for pDPN • Significant improvement in pain intensity in patients with diabetic peripheral neuropathy in large clinical studies • Clinically relevant CYP interactions unlikely to occur	• Late-line treatment agent • Twice-daily formulation • Danger of overdose if tablets not swallowed whole • Requires individual titration • Tapering schedule necessary on discontinuation • Side effects include constipation, nausea, dizziness, headache, and somnolence • Close monitoring of patients for respiratory depression • Caution needed with concomitant use of serotonergic drugs • Contraindicated for use with MAOIs		

*CI* confidence interval, *CYP* cytochrome P450, *ER* extended release, *FDA* Food and Drug Administration, *MAOI* monoamine oxidase inhibitor, *NNH* the number of patients needed to harm for one drop-out due to adverse events, *NNT* estimated number of patients with painful polyneuropathy needed to treat to achieve one patient with a 50 % reduction in pain, *pDPN* painful diabetic peripheral neuropathy, *SSRI* selective serotonin-reuptake inhibitor

### Cannabinoids

Smoked cannabis provides pain relief in HIV-associated neuropathy [89, 90]. However, cannabis oromucosal spray (Sativex®) was not effective in a small study of patients with painful polyneuropathy [91]. Side effects include headache, dizziness, somnolence, dry mouth, constipation, and diarrhea. Regulatory and legal obstacles further complicate the use of cannabinoids for neuropathic pain. The clinical characteristics of cannabis are summarized in Table 5.

**Table 5:** Summary of cannabis as a potential treatment option for diabetic peripheral neuropathy [63–65, 69, 70]

Treatment	Mechanism of action	Advantages	Disadvantages
*Cannabinoids*			
Cannabis	Partial cannabinoid receptor agonist	• Smoked cannabis relieves HIV-associated neuropathy	• Not FDA-approved for pDPN • Side effects include headache, dizziness, somnolence, dry mouth, constipation, and diarrhea • Cannabis spray showed no effect on painful polyneuropathy • Medicolegal and regulatory hurdles • Social stigma

*FDA* Food and Drug Administration, *HIV* human immunodeficiency virus, *pDPN* painful diabetic peripheral neuropathy

### Thioctic Acid

The antioxidant alpha-lipoic acid (thioctic acid) prevents progression of neuropathic impairments and improves neuropathic sensory symptoms including pain [92–94]. Although not all trials were conclusive and some were of poor methodologic quality, meta-analyses demonstrate that intravenous alpha-lipoic acid treatment is associated with significant short-term pain relief and improvements in nerve conduction [92, 95].

## Topical Agents

### Capsaicin

Capsaicin is a transient receptor potential vanilloid type 1 (TRPV1) agonist. Capsaicin creams (0.025–0.075 %) decrease pain in DSPN [96, 97]. The NICE guidelines recommend considering the use of capsaicin cream for patients with localized neuropathic pain who wish to avoid or cannot tolerate oral treatments [64]. The AAN recommends considering capsaicin for the treatment of pDPN (level B recommendation) [69].

A high-concentration 8 % capsaicin patch, administered for 60 minutes by a healthcare professional, provides at least 12 weeks of pain relief in controlled studies of post-herpetic neuralgia (PHN) [98, 99] and HIV-associated DSPN [100]. A meta-analysis of six studies of patients with PHN or painful HIV-associated DSPN confirmed that the high-concentration 8 % capsaicin patch provides significant improvements in pain. The most commonly reported side effects were erythema, burning, or pain localized to the application site [101]. Repeated applications for up to 12 months in patients with painful HIV-associated DSPN provide continued pain relief with reproducible safety and tolerability [102]. The high-concentration 8 % capsaicin patch is FDA approved for PHN, and is approved in the European Union for all forms of peripheral neuropathic pain in patients without diabetes. Two controlled trials of the high-concentration 8 % capsaicin patch in pDPN, NCT01533428 and NCT01478607, are completed, with results from the former showing significantly greater reductions in average daily pain scores for a single 30-minute 8 % capsaicin patch application, maintained for up to 12 weeks, compared with application of a placebo patch (p = 0.018), and results from the latter trial expected by the end of 2015 [103, 104]. The clinical characteristics of capsaicin are summarized in Table 6.

**Table 6:** Summary of topical capsaicin and topical lidocaine as potential treatment options for diabetic peripheral neuropathy [63–65, 69, 70]

Treatment	Mechanism of action	Advantages	Disadvantages	NNT (95 % CI)	NNH (95 % CI)
*Topical agents*		• Minimize unwanted side effects • Suitable for patients who do not wish to take or are intolerant of oral therapies			
Capsaicin cream (0.075 %)	Selective TRPV1 agonist	• Localized pain relief • Low risk of systemic effects • Mildly efficacious	• Side effects include local application-site reactions (burning, stinging, erythema) • Repeated application necessary (3/4 times daily) • Modest pain relief • Recommended duration of use is 8 weeks • Not appropriate for patients with open wounds/sores	11 (5.5–316)	11.5 (8–20)
Capsaicin 8 % patch	Selective TRPV1 agonist	• Localized pain relief • Low risk of systemic effects • Rapid, lasting pain relief • Sustained reductions in pain for 2–3 months with a single brief application (30–60 min) • Can be used as monotherapy or in combination with other treatments	• Not FDA-approved for pDPN • Local application-site reactions (erythema, pain, pruritus, edema, dryness, papules) • Long-term effects of repeated applications on sensation are not yet known		
Lidocaine patch	Blocks voltage-gated sodium channels	• No systemic effects • Well tolerated • Suitable for localized pain • Rapid, localized pain relief • Safe and effective as adjunct to other therapies • Useful in elderly patients or patients with multiple comorbidities	• Not FDA-approved for pDPN • Side effects include mild skin irritations • Label advises that the patch be worn for 12 hours followed by 12-hour treatment break • Patch application may be difficult on some areas (e.g., feet) • Daily patch application may prove cumbersome • Not appropriate for diffuse pain • Not appropriate for patients with open wounds/sores		

*CI* confidence interval, *FDA* Food and Drug Administration, *NNH* the number of patients needed to harm for one drop-out due to adverse events, *NNT* estimated number of patients with painful polyneuropathy needed to treat to achieve one patient with a 50 % reduction in pain, *pDPN* painful diabetic peripheral neuropathy, *PHN* post-herpetic neuralgia, *TRPV1* transient receptor potential vanilloid 1

### Lidocaine Patches

Lidocaine (5 %) medicated patches are approved by the FDA for PHN. Lidocaine blocks sodium channels, so decreasing ectopic discharges. Up to four lidocaine patches may be applied per day. A comparative study indicated that lidocaine (5 %) patches are as effective as pregabalin in reducing neuropathic pain and are well tolerated [105]. Adverse effects include local skin reactions. The AAN recommends that the patch may be considered for the treatment of diabetic neuropathy, giving it a level C recommendation. The clinical characteristics of lidocaine are summarized in Table 6.

### Who Manages DSPN?

There is considerable variability in the role of the healthcare provider who initially diagnoses and ultimately manages DSPN. It can depend on the patient’s presenting symptom or primary complaint, the referral pattern of the primary care physician (PCP), and the patient’s geographic location and access to specialists. As neuropathic symptoms may be the initial complaint of patients with diabetes, they may first present to neurologists, pain specialists, or podiatrists. These specialists must, therefore, recognize DSPN as an initial presenting symptom of diabetes. If the patient is not known to have diabetes, blood screening should include HbA_1c_ levels and an oral glucose tolerance test. As the mainstay of DSPN therapy is glycemic control, general diabetes management should remain within the realm of PCPs, endocrinologists, or diabetes specialists. One benefit of the PCP managing patients with DSPN is that they can also manage the other systemic complications of diabetes beyond neuropathy.

**Fig. 1:**
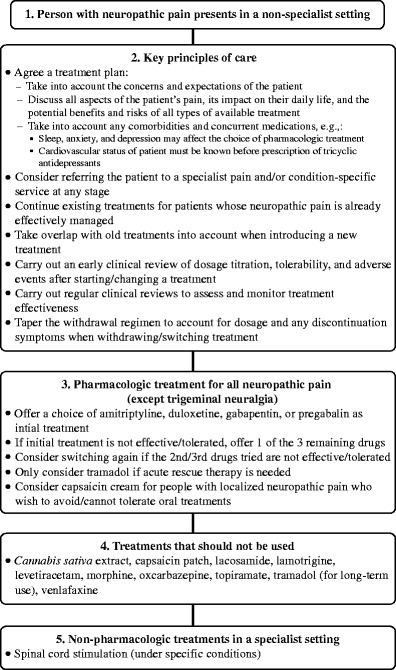
Neuropathic pain pathway adapted from the UK National Institute for Health and Care Excellence [106]

Most PCPs try to manage the complications of diabetes including DSPN until they face certain challenges. The 2013 NICE guidelines recommend referral to a specialist pain service or a condition-specific specialist, such as a neurologist, diabetologist, or oncologist, at any stage if the patient experiences any of the following: severe pain; pain that significantly affects their lifestyle, daily activities, and participation; or deterioration of their underlying health condition (Fig. 1) [106].

In a survey of PCPs and diabetologists, the majority of PCPs did not routinely refer their patients with diabetes to specialty diabetes care [107]. Two-thirds of PCPs reported referring less than a quarter of their patients with diabetes to specialists; reasons for referral included complications with insulin therapy and use of advanced treatment strategies. PCPs were unclear about who was responsible for diabetes management after a specialty referral. Over three-quarters of specialists thought that less than half of PCP practices managed patients with diabetes effectively, through their care or referral to specialists. Reasons were lack of time (73 %), lack of perspective on what to treat and when to refer (69 %), and lack of experience or confidence in designing treatment algorithms (68 %) [107]. The limited number of specialists in rural areas may be a barrier to accessing specialty services. Telemedicine is a possible solution–a simple, cost-effective means of specialists assessing patients in remote locations. A 3D digital optical system is a reliable way of examining diabetic foot ulcers from remote settings, allowing accurate measurements of the wound [108].

There are national variations in referral patterns for patients with DSPN among PCPs. A study in the USA, the UK, and Germany found that for a patient with emerging distal neuropathy, US physicians were most active in terms of questioning, testing, prescribing, and advice giving. US and UK physicians were more likely to refer to a podiatrist than German physicians [109]. There is considerable variability in referral patterns for diabetes management among different European countries [110]. Despite these differences, there was relatively little difference in treatment regimens.

The treatment decision should be tailored to the individual patient, taking into consideration comorbidities, side effects, and drug–drug interactions. Although it is unknown whether treatment preferences differ among different types of specialists, a recent survey found that nearly half of patients with DSPN received nonsteroidal anti-inflammatory drugs (NSAIDs), in spite of their lack of efficacy, and 43 % required opioids, whereas only 27 % were prescribed anticonvulsants and 18 % SNRIs [3]. A large proportion of patients with DSPN appears to be inappropriately treated with NSAIDs, and is over-prescribed opioids. Better education is needed for providers on the treatment of DSPN [111].

Once an ulcer is identified, treatment strategies should shift from symptomatic therapies to more aggressive interventions. Although grade 1 ulcerations (superficial ulcerations) may be managed by a PCP by pressure relief with special footwear, bracing, or casting, grade 2 ulcerations (ulcers penetrating to tendon or capsule) or grade 3 ulcerations (ulcers penetrating to bone or joint) should be referred to a specialist, such as an orthopedic surgeon or podiatrist, for surgical treatment including debridement and possible amputation [112].

### Diabetes Education

Lack of patient education – often due to the limited time during office visits – is a large barrier to active patient participation in glycemic control. Although PCPs are the primary diabetes educators in their practice, specialists appear to rely more on certified diabetes educators (CDEs). However, the proportion of established patients counseled by a CDE at least once yearly appears to be low [107].

### Multidisciplinary Approach

**Fig. 2:**
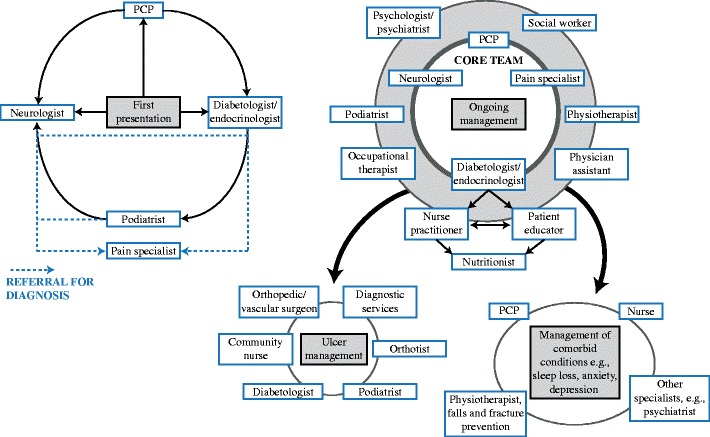
Multidisciplinary team approach to the management of diabetic neuropathic pain/distal symmetric polyneuropathy. PCP—primary care physician

Multidisciplinary teams (Fig. 2) may be the key to reinforcing patient education, particularly when limited time is a hurdle in the outpatient clinic. With smaller practices merging into larger group practices, the role of the multidisciplinary team is growing. In a study of patients with pDPN in a multidisciplinary outpatient setting [113], a PCP referred to a diabetologist-supervised nurse practitioner who then diagnosed and treated all patients according to strict protocols and algorithms. Patients who did not respond to treatment were referred to a specialized outpatient pain clinic. Improvements were found in all pain scores, and in levels of pain interference in sleep, general activity, and mood. The authors concluded that a specialized outpatient clinic for patients with pDPN is an effective healthcare service. Enhancing the role of nursing staff within a practice has also been shown to be particularly helpful [114]. Combining a nurse, patient educator, or both with strict follow-up leads to improvements in patient care and outcomes. Nurses can serve as a liaison between patient and physician, help with patient adherence to treatment and education, and assume some responsibilities of the physician if trained with detailed protocols.

The use of multidisciplinary teams significantly decreased rates of amputation in a hospital in the UK over an 11-year period [115]. Ulcer management should include surgeons, podiatrists, orthotists, clinic-based community nurses, and diabetologists. They should have access to facilities for managing major wounds, including orthopedic or vascular surgery, and diagnostic services, such as microbiology and radiology [116]. As DSPN can be a psychosocial stressor, it is reasonable to include psychologists, social workers, and occupational therapists in the multidisciplinary team.

This multidisciplinary approach also has an economic impact, and with the advent of the Affordable Care Act in the USA, economic pressures may further limit patient access to specialty care. Various solutions have been suggested to address these issues including expanded fixed per-member per-month fees to provide specialty care; compensation models for services such as email, telephone, and curbside consultations; increasing the number of salaried employees of hospital or health systems; and increased peer education [117].

## Conclusions

DSPN is the most common form of diabetic neuropathy and a significant source of patient distress, and an economic and resource burden. Complications include depression, poor sleep, foot ulcers, loss of ambulation, loss of overall function, and amputation. The primary treatment should focus on strict glycemic control and adjustment of modifiable risk factors. There are currently no curative therapies, and symptomatic treatments are recommended by various professional societies. Barriers to effective management include failure to recognize DSPN, particularly when it is asymptomatic. Patients may be in denial of their disease and refuse to actively seek treatment or become noncompliant with medications and interventions.

DSPN is managed in several settings by PCPs, specialists, and nurse practitioners. Management should include patient education, including foot self-examinations, and lifestyle modifications, such as smoking cessation, healthy diet, and exercise. Because strict glycemic control is the mainstay of DSPN treatment, a PCP, endocrinologist, or diabetologist should be involved in care. Referral patterns vary widely according to geographic location, access to services, provider preferences, and physician comfort in managing complex aspects of the disease. The patient should understand the various provider roles and who to address with specific questions. The role of standardized screening tools, the multidisciplinary team, and models utilizing trained nurse practitioners following a neuropathy treatment algorithm are all possible solutions in the streamlining and improvement of future care. Moving forward, physicians should make earlier diagnoses and intervene before significant nerve damage occurs.
